# Acute Acalculous Cholecystitis Revealing Hepatitis A Virus Infection in Children

**DOI:** 10.4103/1319-3767.56098

**Published:** 2009-10

**Authors:** Mounir Arroud, Sara Benmiloud, Bouchra Oudghiri, My Abderrahmane Afifi, Moustapha Hida, Youssef Bouabdallah

**Affiliations:** Departments of Pediatric Surgery, University Hospital Hassan II, Fez, Morocco. E-mail: arroudmounir@hotmail.com; 1 Department of Pediatrics, University Hospital Hassan II, Fez, Morocco; 2 Department of Gastroenterology, University Hospital Hassan II, Fez, Morocco

Sir,

Acute hepatitis A virus (HAV) infection is frequently encountered in developing countries, especially in children. The occurrence of acute acalculous cholecystitis (AAC) during HAV infection is uncommon and it has been exceptionally described in the literature.[1,2]

An 11-year old boy, with an unremarkable medical history, was admitted to the pediatric emergency department with a 4-day history of fever, asthenia, vomiting and abdominal pain. At presentation, the patient had myalgia, jaundice associated with dark urine and pale stool. Physical examination showed body temperature of 38.8°C, scleral and cutaneous icterus and a painful hepatomegaly without associated splenomegaly.

Laboratory studies revealed the following: Hemoglobin, 11.4 mg/dL; white blood cell, 6300/mm^3^; alanine aminotransferase, 1918 U/L (Normal: 7-40 U/L); aspartate aminotransferase, 2953 U/L (Normal: 7-40 U/L); total serum bilirubin, 48 mg/L; with a direct fraction of 27 mg/L, alkaline phosphates 573 U/L (Normal: 38-155 U/L); gamma-glutamyl-transpeptidase, 214 U/L (15-60 U/L); albumin, 3.3 g/dl; prothrombin rate at 62%; C-reactive protein, 66 mg/L (0-5 mg/L).

Abdomen ultrasonography showed a marked thickness of the gallbladder wall evaluated at 11 mm (Normal: ≤2 mm), with a pericholecystic fluid collection and heterogeneous hydrops with no calculi or sludge. The intrahepatic and extrahepatic biliary tracts were normal. Sepsis was suspected and intravenous antibiotics associating clavulanic acid combined with amoxicillin and gentamicin were administered.

The serologic tests showed specific IgM anti-HAV. Viral hepatitis B and C and typhoid diseases were excluded serologically. Bacteriological and parasitological examinations of stools also yielded negative results.

During follow-up, the child remained febrile for three days. At the fifth day, the jaundice and abdominal tenderness regressed. Four days later, the patient was discharged in a stable condition. After one month, abdominal ultrasonography was normal and a significant improvement was also seen in biochemical test results.

HAV infection symbolizes an asymptomatic and benign disease. Cholestasis, relapse and fulminant hepatitis are the three major complications.[3] Pathophysiology of the gallbladder wall thickness remains unclear. Hypoalbuminemia, local extension of the hepatic inflammatory process and direct lesions of the biliary epithelium caused by the virus are the main suggested hypotheses.[4]

Clinical symptoms include fever, jaundice, pain and guarding in the right hypochondrium. Homogeneous or stratified gallbladder wall thickness, greater than 10 mm, noted in ultrasonography examination without any calculous or sludge, confirm the diagnosis of acute acalculous cholecystitis.

The diagnosis of AAC due to HAV infection can be established in the presence of important cytolysis with positive specific IgM anti-HAV. Other possible etiologies of AAC such as salmonellosis, parasitosis of the digestive tract, Kawasaki syndrome and sepsis must be excluded.[2]

AAC as a result of HAV infection is transient and gradually disappears when viremia becomes low. Concurrently, gallbladder wall thickness returns to normal size within a few days. Therefore, patients do not require surgical intervention.

The occurrence of AAC during HAV infection is exceptional. It usually manifests as abdominal pain associated with fever and icterus. This diagnosis should be kept in mind by paediatricians and gastroenterologists to avoid unnecessary invasive procedures.
